# Association Between Serum Folate Concentrations and Dyslipidemia Among Children and Adolescents in Zhejiang Province, China: A Cross-Sectional Study

**DOI:** 10.3390/nu18213556

**Published:** 2026-10-28

**Authors:** Chen Liu, Yi Wang, Lichun Huang, Danting Su, Mengjie He, Dan Han, Yan Wang, Xinhan Zhang, Peiwei Xu, Ronghua Zhang

**Affiliations:** 1 School of Public Health, Hangzhou Medical College, Hangzhou 310059, China; 130232024301@hmc.edu.cn (C.L.);; 2 Zhejiang Provincial Center for Disease Control and Prevention, 3399 Bin Sheng Road, Binjiang District, Hangzhou 310051, Chinarhzhang@cdc.zj.cn (R.Z.)

**Keywords:** folate, dyslipidemia, triglycerides, lipid profile, children and adolescents, China

## Abstract

**Background**: Dyslipidemia can emerge during childhood and persist into adulthood. Although folate is involved in pathways relevant to lipid metabolism, its association with lipid abnormalities in children and adolescents remains unclear. We examined the associations of serum folate concentrations with lipid parameters and dyslipidemia subtypes in this population. **Methods**: This cross-sectional study included 1379 participants aged 6–17 years from the 2024 Zhejiang Provincial Nutrition and Health Surveillance Program, China. Serum folate was measured by liquid chromatography–tandem mass spectrometry. Associations with seven lipid parameters and four dyslipidemia subtypes were evaluated using multivariable regression, with additional restricted cubic spline, subgroup, and sensitivity analyses. **Results**: In fully adjusted models, serum folate was positively associated with TC (β = 0.009, 95% CI: 0.002–0.016), HDL-C (β = 0.007, 95% CI: 0.004–0.010), and ApoA1 (β = 0.003, 95% CI: 0.001–0.005) and inversely associated with TG (β = −0.011, 95% CI: −0.016 to −0.006). Each 1 ng/mL increment was associated with lower odds of hypertriglyceridemia (OR = 0.96, 95% CI: 0.94–0.99) and low HDL-C (OR = 0.91, 95% CI: 0.82–0.99). Quartile and restricted cubic spline analyses supported the hypertriglyceridemia association. **Conclusions**: Higher serum folate concentrations were associated with lower TG, higher HDL-C, and lower odds of hypertriglyceridemia. Evidence for an association with low HDL-C was limited and inconsistent across analyses. Prospective studies are needed to establish temporality.

## 1. Introduction

Cardiovascular disease (CVD) accounts for more deaths globally than any other cause, and abnormal lipid levels constitute a major modifiable contributor to atherosclerotic CVD [1]. Lipid abnormalities may already be present in childhood and include increased concentrations of total cholesterol (TC), triglycerides (TG), or low-density lipoprotein cholesterol (LDL-C), as well as decreased high-density lipoprotein cholesterol (HDL-C) [2]. Although CVD generally manifests in adulthood, adverse lipid profiles may emerge during childhood and adolescence and persist into later life, contributing to the development of atherosclerosis and subsequent cardiovascular disease [3]. The increasing prevalence of childhood overweight and obesity, together with changes in dietary patterns and declining physical activity, has further increased concern about dyslipidemia in children and adolescents [4,5]. Therefore, identifying potentially modifiable nutritional factors associated with lipid abnormalities early in life is of considerable public health importance.

Folate (vitamin B9) is an essential water-soluble B vitamin that serves as an important cofactor in one-carbon metabolism and methylation reactions [6,7]. Although folate has been extensively studied in relation to homocysteine metabolism, increasing evidence indicates that folate status may also be involved in lipid homeostasis through several metabolic pathways [8,9]. One potential link involves hepatic phosphatidylcholine metabolism. Folate-dependent one-carbon metabolism contributes to the methylation of phosphatidylethanolamine to phosphatidylcholine, which is required for the assembly and secretion of very-low-density lipoprotein (VLDL) and the hepatic export of lipids [10,11,12]. Disruption of this process may therefore affect the handling of triglycerides and cholesterol in the circulation [13,14]. Folate status may also influence lipid metabolism through its relationships with oxidative stress, lipid peroxidation, lipoprotein receptor activity, and inflammation [15,16]. These biological links provide a plausible basis for an association between circulating folate concentrations and lipid profiles.

Despite these findings, several important questions remain unresolved. First, evidence regarding the association between serum folate concentrations and dyslipidemia in children and adolescents is limited, particularly in Chinese populations. Second, previous studies have focused predominantly on individual lipid parameters, and relatively few have simultaneously examined multiple lipid components and clinically relevant dyslipidemia subtypes. In addition, whether the associations between serum folate and dyslipidemia show consistent or nonlinear dose–response patterns remains unclear. These gaps are particularly relevant in China, where nationwide mandatory folic acid fortification has not been implemented and circulating folate concentrations may more closely reflect individual dietary intake and nutritional status. Therefore, using data from the 2024 Zhejiang Provincial Nutrition and Health Surveillance Program, we investigated the associations of serum folate concentrations with seven lipid parameters, including TC, TG, HDL-C, LDL-C, apolipoprotein A1 (ApoA1), apolipoprotein B (ApoB), and lipoprotein(a) [Lp(a)], and four dyslipidemia subtypes among children and adolescents aged 6–17 years in Zhejiang Province, China. We also examined potential dose–response relationships. This study aimed to extend the limited epidemiological evidence on serum folate and lipid abnormalities to children and adolescents and to provide further insight into potentially modifiable nutritional factors associated with dyslipidemia early in life.

## 2. Materials and Methods

### 2.1. Study Design and Participants

**Figure 1:**
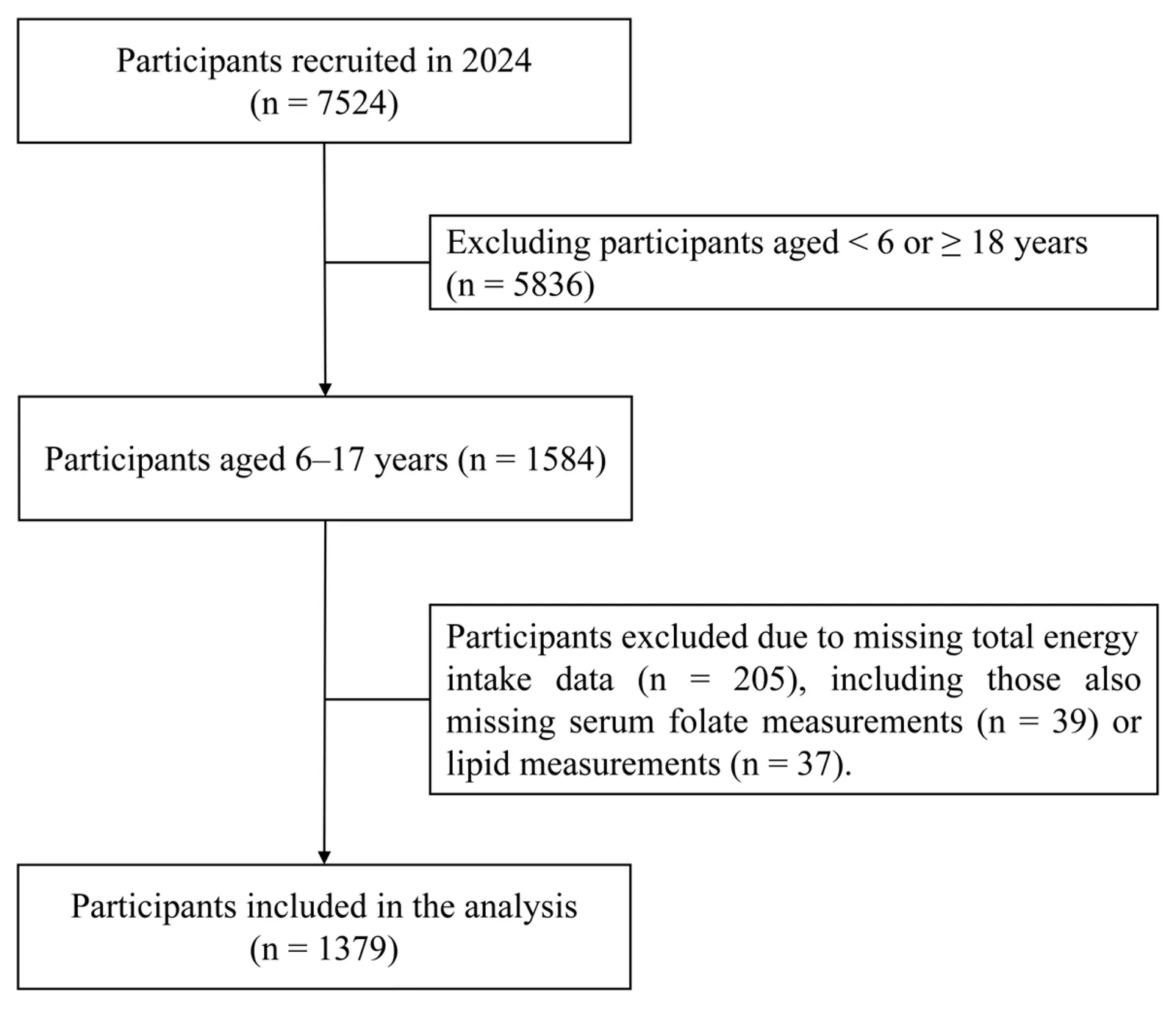
Participant flowchart.

This cross-sectional study was conducted using data from the 2024 Zhejiang Provincial Nutrition and Health Surveillance Program, China. The program adopted a multistage stratified random sampling design to recruit residents who had lived in Zhejiang Province for at least six months. A total of 7524 participants were recruited from 33 counties (cities/districts) across Zhejiang Province. Among them, children and adolescents aged 6–17 years were eligible for the present study. Individuals with missing serum folate concentrations, lipid measurements, or total energy intake were excluded. Finally, 1379 participants were included in the analysis (Figure 1). All participants or their legal guardians provided written informed consent before participation, and standardized questionnaire surveys, physical examinations, and biological sample collection were conducted by trained investigators.

### 2.2. Questionnaire Survey

Information on demographic characteristics, socioeconomic status, health-related behaviors, physical activity, and dietary intake was collected using standardized electronic questionnaires administered through face-to-face interviews by trained investigators. Dietary intake during the previous year was assessed using a validated food frequency questionnaire (FFQ), from which daily energy intake was estimated for subsequent analyses.

### 2.3. Anthropometric Measurements

Anthropometric measurements were performed by trained investigators following standardized protocols during centralized physical examinations. Height and body weight were measured twice, and the average values were used for analysis. Body mass index (BMI) was calculated as weight (kg) divided by the square of height (m^2^). Waist and hip circumferences were also measured according to standardized procedures.

### 2.4. Laboratory Determination

Morning blood samples were obtained from participants after an overnight fast of at least 8 h.

#### 2.4.1. Serum Folate Measurements

Serum folate (vitamin B9) concentrations were measured using liquid chromatography–tandem mass spectrometry (LC–MS/MS; Agilent 1290, Agilent Technologies, Santa Clara, CA, USA, coupled to an AB Sciex 5500 mass spectrometer, AB Sciex LLC., Framingham, MA, USA). For sample preparation, 100 μL of serum was mixed with 300 μL of acetonitrile containing 5-methyltetrahydrofolate-d3 as the internal standard, vortexed for 5 min, and centrifuged at 13,000 rpm for 10 min at 4 °C. The supernatant was transferred to a separate tube, lyophilized, and reconstituted in 100 μL of an aqueous solution containing citric acid, dithiothreitol, and formic acid. Following vortexing for 5 min and centrifugation under the same conditions, 85 μL of the supernatant was transferred to an autosampler vial for analysis. Sample clean-up consisted of protein precipitation, without solid-phase extraction or immunoaffinity columns. Chromatographic separation was performed on an Agilent Eclipse Plus C18 column (150 × 4.6 mm, 5 μm; Agilent Technologies, Inc., Santa Clara, CA, USA) at 40 °C, using a flow rate of 0.6 mL/min and an injection volume of 10 μL. Analytes were detected using electrospray ionization in multiple reaction monitoring mode and quantified using internal standard calibration. The assay had a linear range of 1.13–98.28 ng/mL and a lower limit of quantification of 1.13 ng/mL. Blank samples and pooled serum quality control samples were included in each analytical batch to monitor analytical performance. Intra-assay coefficients of variation ranged from 1.59% to 3.38% across low, medium, and high quality-control concentrations.

#### 2.4.2. Lipid Profile Measurements

Serum lipid profiles were determined using an automatic biochemical analyzer (Beckman AU480, Beckman Coulter, Brea, CA, USA). TC and TG were determined using enzymatic colorimetric methods; HDL-C and LDL-C were determined using homogeneous assays; and ApoA1, ApoB, and Lp(a) were determined using immunoturbidimetric methods.

### 2.5. Definition of Variables

According to the 2022 Expert Consensus on the Diagnosis and Management of Dyslipidemia in Children, hypercholesterolemia was defined as TC ≥ 5.17 mmol/L, hypertriglyceridemia was defined as TG ≥ 1.12 mmol/L for children aged ≤9 years and ≥1.46 mmol/L for those aged ≥10 years, high low-density lipoprotein cholesterol as LDL-C ≥ 3.36 mmol/L, and low high-density lipoprotein cholesterol as HDL-C < 1.03 mmol/L [17]. Age was categorized into 6–11 years and 12–17 years for stratified analyses. Residential area was classified as urban or rural. BMI was calculated as weight in kilograms divided by height in meters squared (kg/m^2^) and was included as a continuous variable in the regression models [18]. For stratified analyses, overweight status was defined according to the Chinese national screening criteria for overweight and obesity among children and adolescents using age- and sex-specific BMI cut-off values. Physical activity was categorized as sufficient (≥1 h/day) or insufficient (<1 h/day) based on the WHO recommendation that children and adolescents aged 5–17 years should engage in an average of at least 60 min/day of moderate-to-vigorous-intensity physical activity [19]. Daily energy intake (kcal/day) and serum methylmalonic acid (MMA) concentrations were included as continuous covariates in the regression analyses.

### 2.6. Statistical Analysis

Continuous variables are expressed as the mean ± standard deviation (SD) for normally distributed variables or as the median (interquartile range, IQR) for non-normally distributed variables. Categorical variables are presented as frequencies and percentages. Characteristics of included and excluded participants were compared using Welch’s *t*-test, the Wilcoxon rank-sum test, or the chi-square test, as appropriate. Comparisons were based on available observations for each variable. We fitted multivariable linear regression models for the seven continuous lipid measures (TC, TG, HDL-C, LDL-C, ApoA1, ApoB, and Lp(a)) and multivariable logistic regression models for the four dyslipidemia subtypes (hypercholesterolemia, hypertriglyceridemia, high LDL-C, and low HDL-C). Linear regression models were assessed using residual-versus-fitted, Q–Q, scale-location, and residual-versus-leverage plots. Serum folate concentration was entered into the models either on its original continuous scale or as a categorical variable defined by quartiles (Q1–Q4), with Q1 as the reference category. Linear trends across quartiles were evaluated by modeling the quartile variable as an ordinal continuous variable (*p* for trend). Three sequential regression models were constructed. Model 1 was unadjusted. Model 2 was adjusted for age, sex, and body mass index (BMI). Model 3 was further adjusted for physical activity, total energy intake, area of residence (urban/rural), and MMA. Sampling weights and complete identifiers for the original sampling strata and primary sampling units were unavailable for this secondary analysis. The reported regression analyses were unweighted and did not incorporate design-based adjustment for stratification or clustering. Odds ratios (ORs) and 95% confidence intervals (CIs) were estimated using profile likelihood methods, and statistical significance was evaluated using likelihood ratio tests (LRTs). Restricted cubic spline (RCS) regression models with four knots placed at the 5th, 35th, 65th, and 95th percentiles of the serum folate distribution were fitted to explore potential nonlinear associations between serum folate concentrations and lipid abnormalities. The RCS analyses incorporated the Model 3 covariates and used the median serum folate concentration as the reference. For lipid abnormalities significantly associated with serum folate in the primary analyses, we performed exploratory analyses within prespecified strata defined by age (6–11 or 12–17 years), sex (male or female), and overweight status (yes or no). Each analysis retained the Model 3 adjustment set except for the corresponding stratification variable. To assess potential effect modification, we added multiplicative interaction terms between serum folate and each stratification variable to the fully adjusted models. Sensitivity analyses examined whether the estimates changed when MMA was omitted from Model 3 or serum homocysteine (Hcy) was included as an additional covariate. Additional sensitivity analyses used natural log-transformed TG and Lp(a) as outcomes, with serum folate retained on its original continuous scale and the same adjustment sets as Models 1–3. To account for multiple testing, the Benjamini–Hochberg procedure was applied separately to the seven continuous lipid outcomes and the four binary dyslipidemia outcomes within each regression model, with serum folate modeled as a continuous variable. All statistical analyses were performed using R software (version 4.5.2; R Foundation for Statistical Computing, Vienna, Austria). All statistical tests were two-sided, and a nominal *p* value < 0.05 or, where applicable, an FDR-adjusted *p* value < 0.05 was considered statistically significant.

## 3. Results

### 3.1. General Characteristics of the Participants

A total of 1379 children and adolescents were included in the present study. The mean age of the participants was 11 ± 3.2 years, and 52% were boys. Overall, 63% of participants were aged 6–11 years, 55% lived in rural areas, and 57% reported engaging in at least 60 min/day of physical activity. The mean BMI was 18.1 ± 3.9 kg/m^2^, and the mean daily energy intake was 1398.2 ± 476.1 kcal/day. The median serum folate concentration was 6.74 ng/mL (IQR: 4.52–10.49 ng/mL), whereas the median MMA and Hcy concentrations were 26.1 ng/mL (IQR: 20.3–34.1 ng/mL) and 1667.9 ng/mL (IQR: 1347.5–2118.1 ng/mL), respectively. Among the participants, 201 (15%) had hypercholesterolemia, 244 (18%) had hypertriglyceridemia, 42 (3.1%) had low HDL-C, and 83 (6.0%) had high LDL-C. Additional baseline characteristics of the study participants are presented in Table 1. Compared with included participants, excluded participants were younger and more likely to live in rural areas and, among those with available measurements, had higher Lp(a) concentrations (all *p* < 0.05). No statistically significant differences were observed in the other characteristics compared (Table S1).

### 3.2. Association Between Serum Folate and Blood Lipid Profiles

The associations between serum folate concentrations and blood lipid profiles are presented in Table 2. In the fully adjusted model (Model 3), serum folate was positively associated with TC (β = 0.009, 95% CI: 0.002–0.016), HDL-C (β = 0.007, 95% CI: 0.004–0.010), and ApoA1 (β = 0.003, 95% CI: 0.001–0.005), and inversely associated with TG (β = −0.011, 95% CI: −0.016 to −0.006). An interquartile-range difference in serum folate (5.97 ng/mL; from 4.52 to 10.49 ng/mL) corresponded to an estimated TG difference of −0.066 mmol/L (95% CI: −0.096 to −0.036) in this model. No statistically significant associations were observed for LDL-C, ApoB, or Lp(a). Estimates were similar across the three models. The associations with TC, TG, HDL-C, and ApoA1 remained statistically significant after Benjamini–Hochberg false discovery rate (FDR) correction (Table S2). Restricted cubic spline analyses indicated nonlinear associations with TG, HDL-C, and ApoA1:TG tended to decrease, whereas HDL-C and ApoA1 tended to increase across the observed range of serum folate concentrations. Serum folate was also positively associated with TC, without clear evidence of nonlinearity. No significant overall associations were observed for LDL-C, ApoB, or Lp(a) (Figure S1).

### 3.3. Multivariable Logistic Regression Analysis of Serum Folate in Relation to Dyslipidemia Subtypes

The associations between serum folate concentrations and dyslipidemia subtypes are presented in Table 3. In Model 3, each 1 ng/mL increment in serum folate was associated with lower odds of hypertriglyceridemia (OR = 0.96, 95% CI: 0.94–0.99; *p* = 0.007). Participants in the highest folate quartile (Q4) also had lower odds of hypertriglyceridemia than those in the lowest quartile (Q1; OR = 0.53, 95% CI: 0.34–0.82; *p* = 0.005), with evidence of a trend across quartiles (*p* for trend = 0.001).

Each 1 ng/mL increment in serum folate was also associated with lower odds of low HDL-C (OR = 0.91, 95% CI: 0.82–0.99; *p* = 0.023), although the trend across folate quartiles was not statistically significant after full adjustment (*p* for trend = 0.175). For hypercholesterolemia, the continuous association was nominally significant (OR = 1.02, 95% CI: 1.00–1.05; *p* = 0.047), but neither the individual quartile comparisons nor the trend test was significant (*p* for trend = 0.314). Serum folate was not significantly associated with high LDL-C in either the continuous analysis (OR = 1.03, 95% CI: 1.00–1.06; *p* = 0.054) or the quartile analyses (*p* for trend = 0.847).

After Benjamini–Hochberg FDR correction, the association with hypertriglyceridemia remained statistically significant, whereas the association with hypercholesterolemia did not. The association with low HDL-C was borderline after correction (FDR-adjusted *p* = 0.046) and should be interpreted cautiously (Table S3).

### 3.4. Dose–Response Relationships Between Serum Folate Concentrations and Dyslipidemia Subtypes

**Figure 2:**
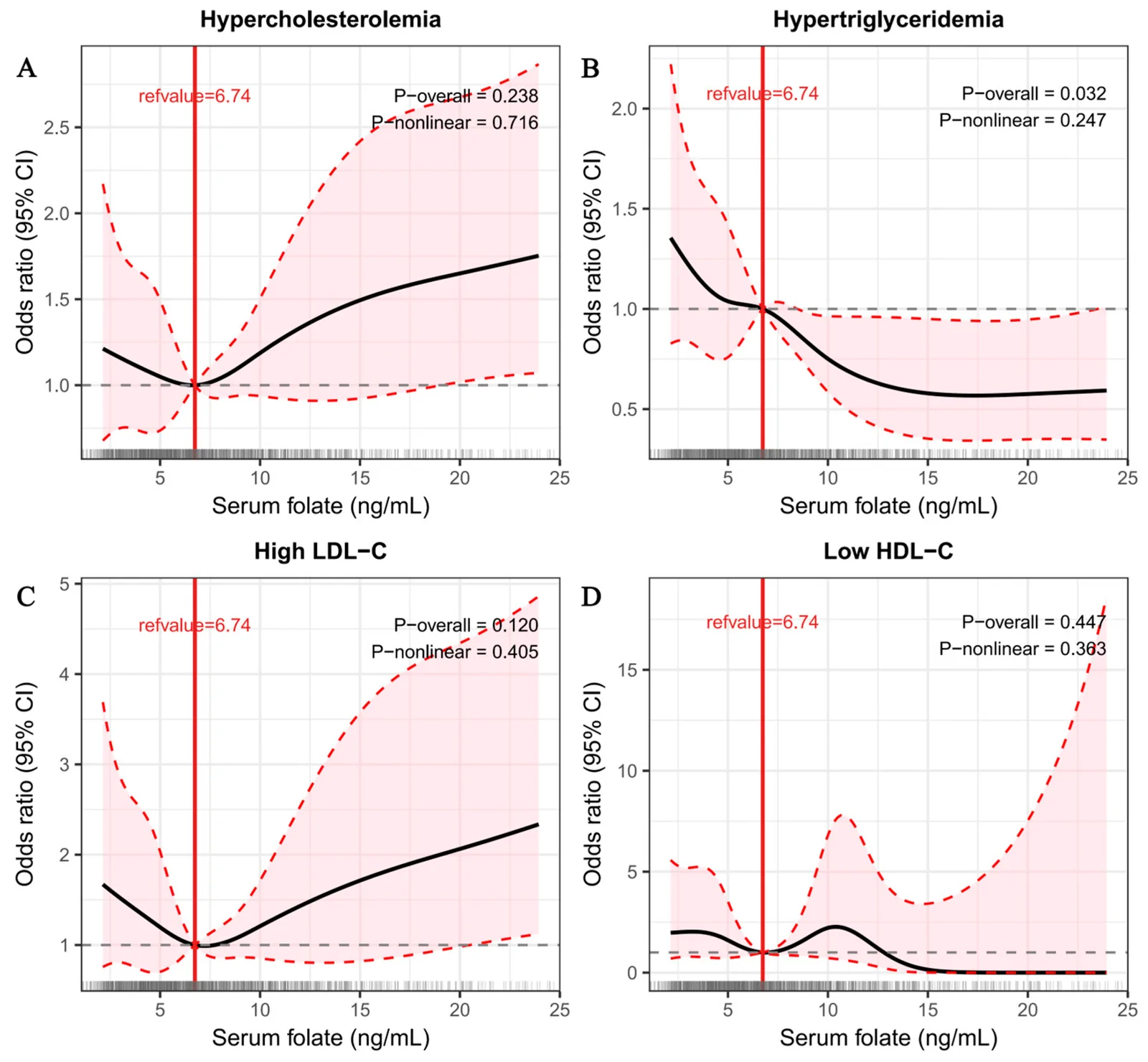
Restricted cubic spline curves for the associations between serum folate concentrations and dyslipidemia subtypes. The models were adjusted for age, sex, BMI, daily physical activity, energy intake, area, and serum MMA. The red vertical line indicates the median serum folate concentration (6.74 ng/mL), which was used as the reference value. The solid black line represents the estimated odds ratio, the shaded area represents the corresponding 95% confidence interval, and the red dashed lines delineate the boundaries of the 95% confidence interval. Gray rug marks along the x-axis indicate the distribution of serum folate concentrations in the analytical sample. *p*-overall indicates the significance of the overall association, and *p*-nonlinearity indicates the significance of the nonlinear component. (**A**) Association between serum folate and hypercholesterolemia. (**B**) Association between serum folate and hypertriglyceridemia. (**C**) Association between serum folate and high LDL-C. (**D**) Association between serum folate and low HDL-C. Abbreviations: BMI, body mass index; MMA, methylmalonic acid; CI, confidence interval; LDL-C, low-density lipoprotein cholesterol; HDL-C, high-density lipoprotein cholesterol.

To further explore the dose–response relationships between serum folate concentrations and dyslipidemia subtypes, restricted cubic spline (RCS) regression models with four knots were constructed after adjustment for the covariates included in Model 3. As shown in Figure 2, the dose–response patterns varied across the four dyslipidemia subtypes. For hypertriglyceridemia (Figure 2B), a significant overall association was observed between serum folate concentrations and the odds of hypertriglyceridemia (*p*-overall = 0.032), with the odds generally decreasing as serum folate concentrations increased. However, no evidence of a significant nonlinear association was observed (*p* for nonlinearity = 0.247), suggesting an approximately linear inverse relationship. No significant overall associations were observed between serum folate concentrations and hypercholesterolemia (Figure 2A; *p*-overall = 0.238), high LDL-C (Figure 2C; *p*-overall = 0.120), or low HDL-C (Figure 2D; *p*-overall = 0.447). In addition, no significant nonlinear associations were detected for any of the four dyslipidemia subtypes (all *p* for nonlinearity > 0.05).

### 3.5. Results of Exploratory Subgroup Analyses

**Figure 3:**
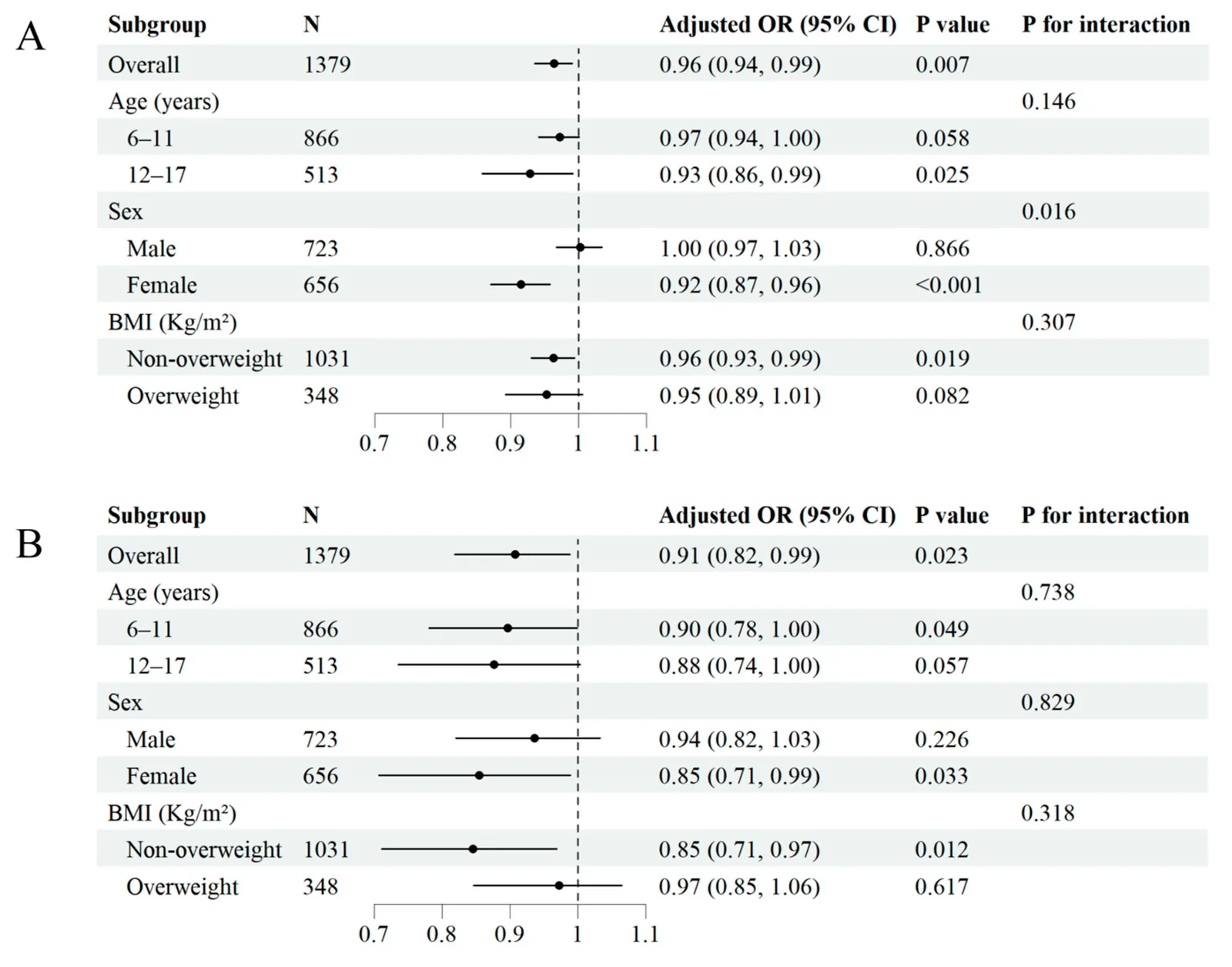
Subgroup analyses of serum folate concentrations in relation to dyslipidemia subtypes. ORs correspond to each 1 ng/mL increment in serum folate. Models were adjusted for age, sex, BMI, physical activity, energy intake, residential area, and MMA, with the variable defining each subgroup excluded from the corresponding adjustment set. Black dots indicate OR estimates, and horizontal lines show 95% CIs. The vertical dashed line denotes OR = 1. (**A**) Hypertriglyceridemia. (**B**) Low HDL-C. *p* values for interaction were obtained by testing multiplicative interaction terms between serum folate and the corresponding stratification variable in the fully adjusted models. Abbreviations: BMI, body mass index; OR, odds ratio; CI, confidence interval; MMA, methylmalonic acid; HDL-C, high-density lipoprotein cholesterol.

Hypertriglyceridemia and low HDL-C were selected for exploratory subgroup analyses based on their significant associations with serum folate in the primary analyses. We examined whether these associations were consistent across strata defined by age, sex, and overweight status. Interaction tests were performed by adding serum folate × stratification variable terms to the fully adjusted models. As shown in Figure 3A, the inverse association between serum folate concentrations and hypertriglyceridemia was generally consistent across age and BMI subgroups. The association was statistically significant among participants aged 12–17 years (OR = 0.93, 95% CI: 0.86–0.99) but not among those aged 6–11 years (OR = 0.97, 95% CI: 0.94–1.00); however, no significant interaction by age was observed (*p* for interaction = 0.146). A significant interaction by sex was detected (*p* for interaction = 0.016), with a significant inverse association observed among females (OR = 0.92, 95% CI: 0.87–0.96) but not among males (OR = 1.00, 95% CI: 0.97–1.03). No significant interaction by BMI was observed (*p* for interaction = 0.307). For low HDL-C (Figure 3B), the inverse association between serum folate concentrations and low HDL-C was observed in the overall population (OR = 0.91, 95% CI: 0.82–0.99). The association was statistically significant among children aged 6–11 years (OR = 0.90, 95% CI: 0.78–1.00) and females (OR = 0.85, 95% CI: 0.71–0.99), as well as those with normal weight (OR = 0.85, 95% CI: 0.71–0.97). However, no significant interactions were observed for age (*p* for interaction = 0.738), sex (*p* for interaction = 0.829), or BMI (*p* for interaction = 0.318). These subgroup analyses were exploratory, and the sex interaction for hypertriglyceridemia should be interpreted cautiously given the multiple comparisons. Only 42 participants had low HDL-C, limiting the precision and reliability of subgroup-specific estimates for this outcome.

### 3.6. Sensitivity Analyses

Several sensitivity analyses were conducted to assess the robustness of the primary findings. First, MMA was removed from the fully adjusted model. The results were largely consistent with those of the primary analyses (Table S4). The positive associations of serum folate with TC, HDL-C, and ApoA1 and the inverse association with TG remained statistically significant after correction for multiple comparisons. For dyslipidemia subtypes, each 1 ng/mL increment in serum folate remained associated with lower odds of hypertriglyceridemia (OR = 0.96, 95% CI: 0.94–0.99) and low HDL-C (OR = 0.91, 95% CI: 0.82–0.99). Participants in the highest folate quartile had lower odds of hypertriglyceridemia than those in the lowest quartile (OR = 0.53, 95% CI: 0.35–0.82; *p* for trend = 0.002). The corresponding quartile association for low HDL-C was not statistically significant (OR = 0.52, 95% CI: 0.18–1.36; *p* for trend = 0.179).

Second, Hcy was additionally included in the fully adjusted model to further assess the robustness of the observed associations after accounting for another one-carbon metabolism-related biomarker (Table S5). Additional adjustment for Hcy yielded estimates broadly similar to those from the primary analyses. Higher serum folate concentrations were associated with higher HDL-C and ApoA1 and lower TG, while associations with LDL-C, ApoB, and Lp(a) were not statistically significant. In the logistic models, higher serum folate was associated with lower odds of hypertriglyceridemia (OR = 0.97, 95% CI: 0.94–1.00) and low HDL-C (OR = 0.89, 95% CI: 0.80–0.98). Removing MMA yielded estimates similar to those of the primary analyses. After additional adjustment for Hcy, the inverse association with hypertriglyceridemia was attenuated and no longer statistically significant after FDR correction, although its direction remained unchanged.

Third, we repeated the fully adjusted analyses using natural log-transformed TG and Lp(a) (Table S6). Serum folate was inversely associated with ln(TG) (β = −0.0120, 95% CI: −0.0164 to −0.0077; *p* < 0.001), whereas its association with ln[Lp(a)] was not statistically significant (β = −0.0031, 95% CI: −0.0121 to 0.0058; *p* = 0.495). These findings were consistent with the inverse association observed for TG and the absence of a statistically significant association for Lp(a) in the primary analyses.

## 4. Discussion

In this cross-sectional study of 1379 children and adolescents in Zhejiang Province, higher serum folate concentrations were associated with lower TG and higher HDL-C and ApoA1 concentrations. Serum folate was also positively associated with TC, although this did not translate into a consistent association with hypercholesterolemia. The inverse association with hypertriglyceridemia was supported by continuous, quartile, and restricted cubic spline analyses, whereas evidence for low HDL-C was less consistent across models. The median serum folate concentration was 6.74 ng/mL, above the 4 ng/mL cutoff used to screen for folate deficiency in China [20]. Exploratory analyses suggested a possible difference by sex in the association with hypertriglyceridemia, which requires confirmation in future studies.

Our results generally agree with earlier epidemiological evidence linking serum folate to lipid profiles, especially TG and HDL-C. In Chinese adults with hypertension, lower serum folate concentrations were associated with higher odds of hypertriglyceridemia and low HDL-C, suggesting a less favorable lipid profile at lower folate levels [21]. Similarly, a retrospective study of adults in Germany found that higher serum folate concentrations were associated with higher HDL-C levels and lower LDL-C levels [22]. In addition, an analysis of the 2011–2016 National Health and Nutrition Examination Survey (NHANES) showed that serum folate concentrations were inversely associated with triglycerides and LDL-C and positively associated with HDL-C [23]. A recent population-based study of adults in Zhejiang Province also reported inverse associations with hypertriglyceridemia and low HDL-C [24]. The agreement across these studies is clearest for TG and HDL-C, whereas findings for cholesterol-related outcomes are less uniform. In our study, serum folate was positively associated with TC as a continuous measure, but the association with hypercholesterolemia did not remain significant after FDR correction and was not supported by the quartile or spline analyses. A study based on the Korean National Health and Nutrition Examination Survey identified nonlinear associations of serum folate with hypercholesterolemia and high LDL-C [25], suggesting that cholesterol-related associations may differ across populations. Differences in age, metabolic background, and the distribution of folate concentrations could contribute to this variation, although these explanations remain uncertain. Most previous studies have focused on adults; by examining both continuous lipid parameters and dyslipidemia subtypes in children and adolescents, our study extends the evidence to an earlier stage of life. Because lipid abnormalities can emerge during childhood and persist into adulthood, identifying factors associated with lipid profiles at this stage may help clarify the early development of dyslipidemia and its potential long-term cardiovascular implications. Although the inverse association between serum folate and TG was statistically significant, the estimated difference across the folate interquartile range was modest (−0.066 mmol/L), and whether this difference is clinically meaningful remains unclear.

The biological basis underlying the observed associations between serum folate and lipid profiles remains unclear, but several plausible pathways may be involved. The relatively consistent associations observed for TG and HDL-C in our study may be related to the role of folate in hepatic lipid handling. Folate-dependent one-carbon metabolism contributes to methyl-group transfer and is closely linked to the synthesis of phosphatidylcholine through the phosphatidylethanolamine N-methyltransferase pathway [7]. Because phosphatidylcholine is an important component required for hepatic VLDL assembly and secretion, disturbances in folate-related one-carbon metabolism could potentially affect the export of triglycerides from the liver and thereby alter circulating TG concentrations [24]. Changes in triglyceride-rich lipoprotein metabolism may also influence HDL-C metabolism, as the turnover of VLDL and other triglyceride-rich particles is closely related to HDL-C concentrations [26,27]. In addition, impaired folate status may increase homocysteine concentrations, with potential consequences for oxidative stress and endothelial function that could further contribute to disturbances in lipid metabolism [28,29]. Although we measured Hcy and considered it in a sensitivity analysis, we did not directly assess phosphatidylcholine synthesis, VLDL metabolism, or the other proposed pathways. These mechanisms therefore remain hypotheses, particularly in children and adolescents.

The spline analysis showed an overall inverse association with hypertriglyceridemia without clear evidence of nonlinearity, although this does not establish a strictly linear relationship. For low HDL-C, the quartile trend and overall spline test were not significant; the small number of cases may have limited precision. No significant overall spline associations were observed for hypercholesterolemia or high LDL-C. Exploratory subgroup analyses suggested that the association with hypertriglyceridemia might differ by sex: it was observed among girls but not boys, and the interaction test was significant. There was no evidence of interaction by age or weight status. For low HDL-C, the interaction tests did not indicate differences by age, sex, or weight status. These subgroup findings require caution because multiple exploratory comparisons were made, and some estimates were imprecise. Larger studies are needed to assess whether sex modifies the folate–hypertriglyceridemia association in children and adolescents. Removing MMA from the adjustment set produced similar estimates. Additional adjustment for Hcy also left the main estimates in the same direction, although the continuous association with hypertriglyceridemia did not remain significant after FDR correction.

The nutritional setting in China is also relevant to the interpretation of our results. While mandatory folic acid fortification is established in some Western countries [30], no equivalent nationwide requirement has been introduced in China [31]. Therefore, serum folate concentrations in our study population may have been more strongly influenced by habitual dietary intake and individual nutritional status, with less contribution from population-wide folic acid fortification. This context may have allowed greater natural variation in circulating folate concentrations to be captured and examined in relation to lipid profiles. However, differences in folate exposure distributions and nutritional backgrounds across populations should be considered when comparing our findings with those from countries with established folic acid fortification programs.

This study has several strengths. First, it included a relatively large community-based sample of children and adolescents in eastern China, a population less studied than adults in research on folate and lipid profiles. Second, directly measured serum folate provided an objective indicator of circulating folate status rather than relying solely on dietary assessment. Third, examining seven continuous lipid parameters and four clinically defined dyslipidemia subtypes allowed assessment of both lipid variation and clinically relevant abnormalities. Fourth, complementary multivariable regression, restricted cubic spline, FDR, exploratory subgroup, and sensitivity analyses assessed the consistency and dose–response patterns of the associations. Adjustment for daily energy intake and MMA, with additional adjustment for Hcy in sensitivity analyses, also allowed potential nutritional and metabolic factors to be considered.

Several limitations should be considered. First, the cross-sectional design precludes establishing the temporal sequence between serum folate concentrations and lipid abnormalities, and causal relationships cannot be inferred. Second, a single serum folate measurement may not capture long-term exposure or nutritional status; repeated measurements or complementary biomarkers could improve assessment. Third, the limited numbers of participants with hypercholesterolemia, high LDL-C, and low HDL-C may have reduced statistical power and the precision of effect estimates, particularly in subgroup and dose–response analyses. Fourth, participants excluded because of missing data differed from included participants in age, residential area, and Lp(a) concentrations, indicating the potential for selection bias. In addition, the analyses did not account for the complex sampling design, which may affect standard errors, confidence intervals, and *p* values. Potential bias arising from unequal selection probabilities could not be assessed with the available information, and the findings should therefore be interpreted cautiously. Finally, despite adjustment for demographic, lifestyle, nutritional, and biochemical factors, residual confounding from unmeasured or inadequately measured factors, including dietary folate intake and folic acid supplement use, cannot be excluded. Prospective studies with repeated measurements, larger samples, and more comprehensive dietary and supplement assessments are warranted to clarify the temporal and potentially causal relationships between folate status and lipid metabolism.

## 5. Conclusions

In this cross-sectional study of children and adolescents in Zhejiang Province, China, higher serum folate concentrations were associated with lower TG concentrations and lower odds of hypertriglyceridemia, with consistent findings across continuous, quartile, and spline analyses. Higher serum folate concentrations were also associated with higher HDL-C and ApoA1 concentrations. The inverse association with low HDL-C was observed when serum folate was modeled continuously but was not supported by the fully adjusted quartile trend or overall spline test; this finding therefore requires cautious interpretation. No consistent associations were observed for hypercholesterolemia or high LDL-C. Because folate and lipid concentrations were measured concurrently, prospective studies are warranted to clarify the temporal and biological relationships between folate status and lipid metabolism in children and adolescents.

## Figures and Tables

**Table 1:** Basic characteristics of the participants (*n* = 1379).

Variables	Overall (1379)
Age (years), [mean ± SD]	10.55 ± 3.15
Age group (years), *n* (%)	
6–11	866 (62.80)
12–17	513 (37.20)
Sex, *n* (%)	
Male	723 (52.43)
Female	656 (47.57)
BMI (Kg/m^2^), [mean ± SD]	18.08 ± 3.93
Area, *n* (%)	
Urban	623 (45.18)
Rural	756 (54.82)
Daily physical activity, *n* (%)	
<60 min/day	591 (42.86)
≥60 min/day	788 (57.14)
Energy intake (kcal/day), [mean ± SD]	1398.16 ± 476.05
Serum folate (ng/mL), [median (IQR)]	6.74 (4.52, 10.49)
MMA (ng/mL), [median (IQR)]	26.08 (20.31, 34.09)
Hcy (ng/mL), [median (IQR)]	1667.9 (1347.50, 2118.10)
TC (mmol/L), [mean ± SD]	4.34 ± 0.81
TG (mmol/L), [median (IQR)]	0.86 (0.60, 1.23)
HDL-C (mmol/L), [mean ± SD]	1.61 ± 0.35
LDL-C (mmol/L), [mean ± SD]	2.33 ± 0.61
ApoA1 (g/L), [mean ± SD]	1.51 ± 0.22
ApoB (g/L), [mean ± SD]	0.70 ± 0.15
Lp(a) (mg/dL), [median (IQR)]	14.20 (7.67, 29.93)
Hypercholesterolemia, *n* (%)	201 (14.58)
Hypertriglyceridemia, *n* (%)	244 (17.69)
Low HDL-C, *n* (%)	42 (3.05)
High LDL-C, *n* (%)	83 (6.02)

Abbreviations: SD, standard deviation; IQR, interquartile range; BMI, body mass index; TC, total cholesterol; TG, triglycerides; HDL-C, high-density lipoprotein cholesterol; LDL-C, low-density lipoprotein cholesterol; ApoA1, apolipoprotein A1; ApoB, apolipoprotein B; Lp(a), lipoprotein(a); MMA, methylmalonic acid; Hcy, homocysteine.

**Table 2:** Associations between serum folate concentrations and blood lipid profiles.

Outcome	Model 1		Model 2		Model 3	
	β (95% CI)	*p*	β (95% CI)	*p*	β (95% CI)	*p*
TC	0.012 (0.005, 0.018)	<0.001	0.009 (0.002, 0.016)	0.011	0.009 (0.002, 0.016)	0.011
TG	−0.014 (−0.020, −0.009)	<0.001	−0.011 (−0.016, −0.006)	<0.001	−0.011 (−0.016, −0.006)	<0.001
HDL-C	0.011 (0.008, 0.014)	<0.001	0.007 (0.004, 0.010)	<0.001	0.007 (0.004, 0.010)	<0.001
LDL-C	0.003 (−0.002, 0.008)	0.223	0.004 (−0.001, 0.009)	0.122	0.004 (−0.001, 0.009)	0.126
ApoA1	0.005 (0.003, 0.007)	<0.001	0.003 (0.001, 0.005)	0.005	0.003 (0.001, 0.005)	0.005
ApoB	0.000 (−0.001, 0.001)	0.983	0.000 (−0.001, 0.002)	0.601	0.000 (−0.001, 0.002)	0.606
Lp(a)	−0.108 (−0.323, 0.107)	0.326	−0.101 (−0.327, 0.125)	0.381	−0.098 (−0.325, 0.128)	0.395

Abbreviations: CI, confidence interval; BMI, body mass index; MMA, methylmalonic acid; FDR, false discovery rate; TC, total cholesterol; TG, triglycerides; HDL-C, high-density lipoprotein cholesterol; LDL-C, low-density lipoprotein cholesterol; ApoA1, apolipoprotein A1; ApoB, apolipoprotein B; Lp(a), lipoprotein(a). Model 1 was unadjusted. Model 2 was adjusted for age, sex, and BMI. Model 3 was additionally adjusted for daily physical activity, energy intake, area, and MMA. *p* values shown are unadjusted for multiple testing; Benjamini–Hochberg FDR-adjusted *p* values are provided in Table S2.

**Table 3:** Multivariable logistic regression analysis of serum folate concentrations in relation to dyslipidemia subtypes.

Characteristics	Model 1		Model 2		Model 3	
	OR (95% CI)	*p*	OR (95% CI)	*p*	OR (95% CI)	*p*
Hypercholesterolemia Serum folate per 1 ng/mL increment	1.03 (1.00, 1.05)	0.019	1.02 (1.00, 1.05)	0.045	1.02 (1.00, 1.05)	0.047
Serum folate quartile						
Q1 (*n* = 346)	1.00 (Reference)		1.00 (Reference)		1.00 (Reference)	
Q2 (*n* = 345)	0.80 (0.51, 1.24)	0.319	0.77 (0.49, 1.20)	0.250	0.72 (0.46, 1.13)	0.159
Q3 (*n* = 343)	0.96 (0.63, 1.48)	0.864	0.91 (0.58, 1.41)	0.657	0.89 (0.57, 1.38)	0.599
Q4 (*n* = 345)	1.30 (0.86, 1.95)	0.210	1.20 (0.78, 1.86)	0.414	1.16 (0.75, 1.80)	0.499
*p* for trend		0.136		0.290		0.314
Hypertriglyceridemia Serum folate per 1 ng/mL increment	0.97 (0.94, 0.99)	0.014	0.97 (0.94, 0.99)	0.008	0.96 (0.94, 0.99)	0.007
Serum folate quartile						
Q1 (*n* = 346)	1.00 (Reference)		1.00 (Reference)		1.00 (Reference)	
Q2 (*n* = 345)	1.00 (0.69, 1.45)	0.985	0.96 (0.66, 1.40)	0.832	0.98 (0.67, 1.43)	0.923
Q3 (*n* = 343)	0.76 (0.51, 1.11)	0.157	0.70 (0.47, 1.05)	0.085	0.70 (0.46, 1.04)	0.078
Q4 (*n* = 345)	0.60 (0.40, 0.89)	0.012	0.54 (0.35, 0.84)	0.006	0.53 (0.34, 0.82)	0.005
*p* for trend		0.005		0.002		0.001
High LDL-C Serum folate per 1 ng/mL increment	1.02 (0.99, 1.05)	0.111	1.03 (1.00, 1.06)	0.050	1.03 (1.00, 1.06)	0.054
Serum folate quartile						
Q1 (*n* = 346)	1.00 (Reference)		1.00 (Reference)		1.00 (Reference)	
Q2 (*n* = 345)	0.56 (0.28, 1.06)	0.082	0.59 (0.30, 1.12)	0.113	0.57 (0.29, 1.10)	0.098
Q3 (*n* = 343)	0.72 (0.39, 1.32)	0.296	0.77 (0.41, 1.45)	0.425	0.78 (0.41, 1.46)	0.436
Q4 (*n* = 345)	0.88 (0.49, 1.57)	0.664	1.00 (0.53, 1.87)	0.998	0.99 (0.53, 1.87)	0.987
*p* for trend		0.812		0.868		0.847
Low HDL-C Serum folate per 1 ng/mL increment	0.86 (0.78, 0.94)	<0.001	0.92 (0.83, 0.99)	0.034	0.91 (0.82, 0.99)	0.023
Serum folate quartile						
Q1 (*n* = 346)	1.00 (Reference)		1.00 (Reference)		1.00 (Reference)	
Q2 (*n* = 345)	0.34 (0.13, 0.77)	0.015	0.42 (0.16, 0.98)	0.057	0.42 (0.16, 1.00)	0.058
Q3 (*n* = 343)	0.44 (0.19, 0.95)	0.044	0.68 (0.28, 1.53)	0.366	0.62 (0.25, 1.42)	0.274
Q4 (*n* = 345)	0.29 (0.10, 0.69)	0.008	0.55 (0.19, 1.40)	0.234	0.52 (0.18, 1.35)	0.204
*p* for trend		0.005		0.224		0.175

Abbreviations: OR, odds ratio; CI, confidence interval; BMI, body mass index; MMA, methylmalonic acid; LDL-C, low-density lipoprotein cholesterol; HDL-C, high-density lipoprotein cholesterol. Model 1 was unadjusted. Model 2 was adjusted for age, sex, and BMI. Model 3 was additionally adjusted for daily physical activity, energy intake, area, and MMA. Serum folate concentrations were analyzed as continuous variables (per 1 ng/mL increment) and categorized into quartiles (Q1–Q4), with the lowest quartile (Q1) serving as the reference group. *p* for trend was calculated by coding serum folate quartiles as 1, 2, 3, and 4 in the logistic regression model.

## Data Availability

The data presented in this study are available upon request from the corresponding author (the data are not publicly available due to privacy restrictions).

## Supplementary Materials

The following supporting information can be downloaded at https://www.mdpi.com/article/10.3390/nu18213556/s1, Figure S1: Dose–response relationships between serum folate and continuous lipid parameters; Table S1: Comparison of characteristics between included and excluded participants; Table S2: Associations between serum folate concentrations and blood lipid profiles; Table S3: Associations between serum folate concentrations and dyslipidemia subtypes; Table S4: Sensitivity analysis of the associations between serum folate concentrations and lipid profiles after removing serum methylmalonic acid (MMA); Table S5: Sensitivity analysis of the associations between serum folate concentrations and lipid profiles after additionally adjusting for serum homocysteine (Hcy); Table S6: Sensitivity analyses of the associations between serum folate concentrations and natural log-transformed TG and Lp(a).
