# Supplementary material for: Association Between Serum Folate Concentrations and Dyslipidemia Among Children and Adolescents in Zhejiang Province, China: A Cross-Sectional Study
Source: Nutrients. 2026 Oct 28;18(21):3556. doi: 10.3390/nu18213556 (PMC13764756; doi:10.3390/nu18213556)
Supplement: Supplementary file 1 [file nutrients-18-03556-s001.zip › Table S1.pdf]

Table S1. Comparison of characteristics between included and excluded participants.

Characteristic	Included participants	Available N	Excluded participants	Available N	P
Age (years)	10.55 ± 3.15	1379	10.07 ± 3.01	205	0.035
Male	723 (52.4%)	1379	96 (46.8%)	205	0.134
Female	656 (47.6%)	1379	109 (53.2%)	205	
BMI (kg/m²)	18.08 ± 3.93	1379	18.47 ± 4.27	205	0.230
<60 min/day	591 (42.9%)	1379	88 (42.9%)	205	0.985
⩾60 min/day	788 (57.1%)	1379	117 (57.1%)	205	
Urban	623 (45.2%)	1379	74 (36.1%)	205	0.015
Rural	756 (54.8%)	1379	131 (63.9%)	205	
Serum folate (ng/mL)	6.74 (4.52, 10.49)	1379	7.36 (4.98, 10.06)	166	0.278
MMA	26.08 (20.31, 34.09)	1379	25.65 (20.60, 34.90)	205	0.693
TC	4.34 ± 0.81	1379	4.31 ± 0.71	168	0.520
TG	0.86 (0.60, 1.23)	1379	0.88 (0.60, 1.23)	168	0.729
HDL-C	1.61 ± 0.35	1379	1.58 ± 0.33	168	0.307
LDL-C	2.33 ± 0.61	1379	2.34 ± 0.57	168	0.872
ApoA1	1.51 ± 0.22	1379	1.50 ± 0.21	168	0.415
ApoB	0.70 ± 0.15	1379	0.70 ± 0.15	168	0.564
LP(a)	14.20 (7.67, 29.92)	1379	16.48 (9.21, 34.55)	168	0.029

Data are presented as mean ± standard deviation, median (25th, 75th percentiles), or n (%), as appropriate. Between-group comparisons were performed using Welch’s t-test for age, BMI, TC, HDL-C, LDL-C, ApoA1, and ApoB; the Wilcoxon rank-sum test for serum folate, TG, and Lp(a); and the chi-square test for categorical variables. Comparisons were based on available observations for each variable, and percentages were calculated using non-missing observations within each group. Among excluded participants, serum folate measurements were available for 166 participants and lipid measurements for 168. P values are nominal and were not adjusted for multiple testing. Abbreviations: BMI, body mass index; TC, total cholesterol; TG, triglycerides; HDL-C, high-density lipoprotein cholesterol; LDL-C, low-density lipoprotein cholesterol; ApoA1, apolipoprotein A1; ApoB, apolipoprotein B; Lp(a), lipoprotein(a). MMA, methylmalonic acid.
