# Supplementary material for: Association Between Serum Folate Concentrations and Dyslipidemia Among Children and Adolescents in Zhejiang Province, China: A Cross-Sectional Study
Source: Nutrients. 2026 Oct 28;18(21):3556. doi: 10.3390/nu18213556 (PMC13764756; doi:10.3390/nu18213556)
Supplement: Supplementary file 1 [file nutrients-18-03556-s001.zip › Table S6.pdf]

Table S6. Sensitivity analyses of the associations between serum folate concentrations and natural log-transformed TG and Lp(a).

Outcome	Model 1		Model 2		Model 3	
	β (95% CI)	p	β (95% CI)	p	β (95% CI)	p
ln(TG)	−0.016 (−0.020, −0.012)	<0.001	−0.012 (−0.017, −0.008)	<0.001	−0.012 (−0.016, −0.008)	<0.001
ln[Lp(a)]	−0.002 (−0.011, 0.006)	0.613	−0.003 (−0.012, 0.006)	0.485	−0.003 (−0.012, 0.006)	0.495

TG and Lp(a) were natural log-transformed before analysis. β coefficients represent differences in the natural logarithm of the outcome per 1 ng/mL higher serum folate concentration. Model 1 was unadjusted. Model 2 was adjusted for age, sex, and BMI. Model 3 was additionally adjusted for daily physical activity, energy intake, area, serum methylmalonic acid (MMA). P values are nominal and were not adjusted for multiple testing.
