# Supplementary material for: Association Between Serum Folate Concentrations and Dyslipidemia Among Children and Adolescents in Zhejiang Province, China: A Cross-Sectional Study
Source: Nutrients. 2026 Oct 28;18(21):3556. doi: 10.3390/nu18213556 (PMC13764756; doi:10.3390/nu18213556)
Supplement: Supplementary file 1 [file nutrients-18-03556-s001.zip › Table S5.pdf]

Table S5. Sensitivity analysis of the associations between serum folate concentrations and lipid profiles after additionally adjusting for serum homocysteine (Hcy)

Panel A. Blood lipid profiles

Outcome	Model 3		
	β (95% CI)	p	FDRp
TC	0.008 (0.001, 0.015)	0.029	0.050
TG	-0.009(-0.015, -0.004)	0.001	0.004
HDL-C	0.006 (0.003, 0.009)	0.000	0.000
LDL-C	0.003 (-0.002, 0.009)	0.236	0.330
ApoA1	0.002 (0.000, 0.004)	0.024	0.050
ApoB	0.000 (-0.001, 0.002)	0.750	0.750
Lp(a)	-0.125 (-0.356, 0.107)	0.291	0.339

Panel B. Dyslipidemia subtypes

Characteristics	Model 3		
	OR (95% CI)	p	FDRp
Hypercholesterolemia<br>Serum folate per 1 ng/mL increment	1.02 (1.00, 1.04)	0.093	0.093
Serum folate quartile			
Q1 (n = 346)	1.00 (Reference)	-	
Q2 (n = 345)	0.67 (0.42, 1.06)	0.092	
Q3 (n = 343)	0.81 (0.52, 1.28)	0.370	
Q4 (n = 345)	1.05 (0.67, 1.65)	0.842	
p for trend		0.549	
Hypertriglyceridemia<br>Serum folate per 1 ng/mL increment	0.97 (0.94, 1.00)	0.031	0.061
Serum folate quartile			
Q1 (n = 346)	1.00 (Reference)	-	
Q2 (n = 345)	1.06 (0.72, 1.57)	0.754	
Q3 (n = 343)	0.76 (0.50, 1.16)	0.204	
Q4 (n = 345)	0.60 (0.38, 0.94)	0.026	
p for trend		0.010	
High LDL-C<br>Serum folate per 1 ng/mL increment	1.03 (1.00, 1.06)	0.075	0.093
Serum folate quartile			
Q1 (n = 346)	1.00 (Reference)	-	
Q2 (n = 345)	0.53 (0.26, 1.04)	0.068	
Q3 (n = 343)	0.71 (0.37, 1.36)	0.307	
Q4 (n = 345)	0.90 (0.47, 1.73)	0.752	
p for trend		0.965	
Low HDL-C<br>Serum folate per 1 ng/mL increment	0.89 (0.80, 0.98)	0.011	0.044
Serum folate quartile			
Q1 (n = 346)	1.0000 (Reference)	-	
Q2 (n = 345)	0.3813 (0.1432, 0.9120)	0.038	
Q3 (n = 343)	0.5477 (0.2191, 1.2883)	0.178	
Q4 (n = 345)	0.4431 (0.1460, 1.1972)	0.124	
p for trend		0.110	

The sensitivity analysis was conducted after additionally adjusting the fully adjusted model for serum Hcy.The model was adjusted for age, sex, BMI, daily physical activity, energy intake, and area, MMA, and Hcy. For blood lipid profiles, β coefficients and 95% confidence intervals (CIs) were estimated using multivariable linear regression. For dyslipidemia subtypes, odds ratios (ORs) and 95% CIs were estimated using multivariable logistic regression. Serum folate was analyzed as a continuous variable per 1 ng/mL increment and as quartiles, with the lowest quartile (Q1) as the reference. P values for the seven lipid parameters were corrected for multiple comparisons using the Benjamini–Hochberg false discovery rate (FDR) procedure. P for trend was calculated by modeling serum folate quartiles as an ordinal variable.
