# Supplementary material for: Association Between Serum Folate Concentrations and Dyslipidemia Among Children and Adolescents in Zhejiang Province, China: A Cross-Sectional Study
Source: Nutrients. 2026 Oct 28;18(21):3556. doi: 10.3390/nu18213556 (PMC13764756; doi:10.3390/nu18213556)
Supplement: Supplementary file 1 [file nutrients-18-03556-s001.zip › Table S4.pdf]

Table S4. Sensitivity analysis of the associations between serum folate concentrations and lipid profiles after removing serum methylmalonic acid (MMA)

Panel A. Blood lipid profiles

Outcome	Model 3		
	**β (95% CI)**	**p**	**FDRp**
TC	0.009 (0.002, 0.016)	0.011	0.018
TG	-0.011 (-0.017, -0.006)	0.000	0.000
HDL-C	0.007 (0.004, 0.010)	0.000	0.000
LDL-C	0.004 (-0.001, 0.009)	0.122	0.171
ApoA1	0.003 (0.001, 0.005)	0.005	0.012
ApoB	0.000 (-0.001, 0.002)	0.598	0.598
Lp(a)	-0.099(-0.325, 0.127)	0.391	0.456

Panel B. Dyslipidemia subtypes

Characteristics	Model 3		
	**OR (95% CI)**	**p**	**FDRp**
Hypercholesterolemia<br>Serum folate per 1 ng/mL increment	1.02 (1.00, 1.05)	0.044	0.051
Serum folate quartile			
Q1 (n = 346)	1.00 (Reference)	-	
Q2 (n = 345)	0.74 (0.47, 1.16)	0.186	
Q3 (n = 343)	0.89 (0.57, 1.38)	0.600	
Q4 (n = 345)	1.17 (0.76, 1.82)	0.477	
p for trend		0.315	
Hypertriglyceridemia<br>Serum folate per 1 ng/mL increment	0.96 (0.94, 0.99)	0.007	0.029
Serum folate quartile			
Q1 (n = 346)	1.00 (Reference)	-	
Q2 (n = 345)	0.96 (0.66, 1.40)	0.835	
Q3 (n = 343)	0.70 (0.47, 1.04)	0.080	
Q4 (n = 345)	0.53 (0.35, 0.82)	0.005	
p for trend		0.002	
High LDL-C<br>Serum folate per 1 ng/mL increment	1.03 (1.00, 1.06)	0.051	0.051
Serum folate quartile			
Q1 (n = 346)	1.00 (Reference)	-	
Q2 (n = 345)	0.58 (0.29, 1.11)	0.106	
Q3 (n = 343)	0.77 (0.40, 1.45)	0.430	
Q4 (n = 345)	1.00 (0.53, 1.88)	0.994	
p for trend		0.845	
Low HDL-C<br>Serum folate per 1 ng/mL increment	0.91 (0.82, 0.99)	0.024	0.048
Serum folate quartile			
Q1 (n = 346)	1.00 (Reference)	-	
Q2 (n = 345)	0.42 (0.16, 0.99)	0.058	
Q3 (n = 343)	0.63 (0.26, 1.44)	0.284	
Q4 (n = 345)	0.52 (0.18, 1.36)	0.205	
p for trend		0.179	

The sensitivity analysis was conducted after removing serum MMA from the fully adjusted model. The model was adjusted for age, sex, BMI, daily physical activity, energy intake, and area. For blood lipid profiles, β coefficients and 95% confidence intervals (CIs) were estimated using multivariable linear regression. For dyslipidemia subtypes, odds ratios (ORs) and 95% CIs were estimated using multivariable logistic regression. Serum folate was analyzed as a continuous variable per 1 ng/mL increment and as quartiles, with the lowest quartile (Q1) as the reference. P values for the seven lipid parameters were corrected for multiple comparisons using the Benjamini–Hochberg false discovery rate (FDR) procedure. P for trend was calculated by modeling serum folate quartiles as an ordinal variable.
