# Supplementary material for: Association Between Serum Folate Concentrations and Dyslipidemia Among Children and Adolescents in Zhejiang Province, China: A Cross-Sectional Study
Source: Nutrients. 2026 Oct 28;18(21):3556. doi: 10.3390/nu18213556 (PMC13764756; doi:10.3390/nu18213556)
Supplement: Supplementary file 1 [file nutrients-18-03556-s001.zip › Table S3.pdf]

Table S3. Associations between serum folate concentrations and dyslipidemia subtypes

Outcome	Model 1		Model 2		Model 3	
	**OR (95% CI)**	**FDRp**	**OR (95% CI)**	**FDRp**	**OR (95% CI)**	**FDRp**
hypercholesterolemia	1.03 (1.00, 1.05)	0.025	1.02 (1.00, 1.05)	0.050	1.02 (1.00, 1.05)	0.054
Hypertriglyceridemia	0.97 (0.94, 0.99)	0.025	0.97 (0.94, 0.99)	0.034	0.96 (0.94, 0.99)	0.027
High LDL-C	1.02 (0.99, 1.05)	0.111	1.03 (1.00, 1.06)	0.050	1.03 (1.00, 1.06)	0.054
Low HDL-C	0.86 (0.78, 0.94)	0.001	0.92 (0.83, 0.99)	0.050	0.91 (0.82, 0.99)	0.047

Model 1 was unadjusted. Model 2 was adjusted for age, sex, and BMI. Model 3 was additionally adjusted for daily physical activity, energy intake, area, serum methylmalonic acid (MMA). P values were corrected for multiple comparisons using the Benjamini–Hochberg false discovery rate (FDR) procedure.
