# Supplementary material for: Association Between Serum Folate Concentrations and Dyslipidemia Among Children and Adolescents in Zhejiang Province, China: A Cross-Sectional Study
Source: Nutrients. 2026 Oct 28;18(21):3556. doi: 10.3390/nu18213556 (PMC13764756; doi:10.3390/nu18213556)
Supplement: Supplementary file 1 [file nutrients-18-03556-s001.zip › Table S2.pdf]

Table S2. Associations between serum folate concentrations and blood lipid profiles

Outcome	Model 1		Model 2		Model 3	
	**β (95% CI)**	**FDRp**	**β (95% CI)**	**FDRp**	**β (95% CI)**	**FDRp**
TC	0.012 (0.005, 0.018)	0.001	0.009 (0.002, 0.016)	0.019	0.009 (0.002, 0.016)	0.019
TG	−0.014 (−0.020, −0.009)	<0.001	−0.011 (−0.016, −0.006)	<0.001	−0.011 (−0.016, −0.006)	<0.001
HDL-C	0.011 (0.008, 0.014)	<0.001	0.007 (0.004, 0.010)	<0.001	0.007 (0.004, 0.010)	<0.001
LDL-C	0.003 (−0.002, 0.008)	0.313	0.004 (−0.001, 0.009)	0.170	0.004 (−0.001, 0.009)	0.176
ApoA1	0.005 (0.003, 0.007)	<0.001	0.003 (0.001, 0.005)	0.012	0.003 (0.001, 0.005)	0.012
ApoB	0.000(−0.001, 0.001)	0.983	0.000 (−0.001, 0.002)	0.601	0.000 (−0.001, 0.002)	0.606
Lp(a)	−0.108(−0.323, 0.107)	0.380	−0.101 (−0.327, 0.125)	0.445	−0.098 (−0.325, 0.128)	0.460

Model 1 was unadjusted. Model 2 was adjusted for age, sex, and BMI. Model 3 was additionally adjusted for daily physical activity, energy intake, area, serum methylmalonic acid (MMA). P values were corrected for multiple comparisons using the Benjamini–Hochberg false discovery rate (FDR) procedure.
